# A self-efficacy-enhancing intervention for Chinese patients after total hip arthroplasty: study protocol for a randomized controlled trial with 6-month follow-up

**DOI:** 10.1186/s13018-021-02689-8

**Published:** 2022-01-04

**Authors:** Bo Deng, Yumei Chen, Ya Meng, Yiheng Zhang, Xingxian Tan, Xiaohong Zhou, Meifen Zhang

**Affiliations:** 1 Nursing Department, Sun Yat-sen University Cancer Center, Guangzhou, Guangdong China; 2 Nursing Department, Foshan Hospital of Traditional Chinese Medicine, Foshan, Guangdong China; 3 School of Nursing, Sun Yat-sen University, 74, Zhongshan 2nd Rd, Guangzhou, 510080 Guangdong China; 4 Department of Orthopedics, Foshan Hospital of Traditional Chinese Medicine, Foshan, Guangdong China

**Keywords:** Total hip arthroplasty, Self-efficacy, Exercise adherence, Rehabilitation

## Abstract

**Background:**

Total hip arthroplasty (THA) is a common and effective surgical method for advanced hip arthritis. Rehabilitation exercises are important to improve joint function after THA and are usually conducted in a home-based program. Poor patient adherence limits improvements in pain and function, affecting quality of life. The increasing use of THA in the aging Chinese population underscores the need to develop strategies that maximize functional outcomes. The purpose of this pilot study is to develop and assess the feasibility of a self-efficacy-enhancing intervention (SEEI) to improve exercise adherence in patients undergoing THA.

**Methods:**

This single-blinded, parallel, randomized control trial will recruit 150 patients after THA and randomly assign them to an intervention or control group using computer-generated block randomization. The control group will receive usual care using evidence-based guidelines. The intervention group will receive the 6-month SEEI comprising personalized exercise guidance and self-efficacy education delivered using one face-to-face education session and four telephone consultations, supplemented by written materials. Participants are encouraged to build confidence in their own abilities, set rehabilitation goals, and self-monitor their physical exercise.

**Results:**

Assessments will be conducted at baseline and 1, 3, and 6 months postsurgery. The outcome indicators are exercise adherence, physical function, anxiety and depression, self-efficacy of rehabilitation, joint function, and quality of life.

**Conclusions:**

This study will test a theory-based intervention program to improve self-efficacy in rehabilitation, which may significantly impact out-of-hospital rehabilitation. The results will provide evidence to inform the postoperative recovery of patients undergoing THA or similar procedures.

**Trial registration:**

Chinese Clinical Trials Registry, ChiCTR2000029422, registered on 31 January 2020

## Background

Total hip arthroplasty (THA) is a major surgical procedure performed worldwide [1]. Approximately 400,000 THAs are performed annually in China [2], and this is growing rapidly due to the high level of patient needs and the challenges of an aging population [3, 4]. THA is a cost-effective intervention that reduces or even eliminates pain [5]. Despite these encouraging facts, differences remain in patient self-reported functional improvement after THA [6, 7]. Some studies have reported significant positive correlations between the surgical effects in patients after hip/knee arthroplasty and exercise adherence [8–11]. Exercise adherence is indeed a fundamental determinant to the success of hip arthroplasty, as plenty of new technologies and integrated pathways aim to enhance [12]. Home exercise adherence is usually high in the early stage but gradually decreases over time [13, 14]. As poor adherence to home exercise is common and affects therapeutic effectiveness and healthcare costs [15], there is an urgent need for economic, achievable, and effective interventions to improve home exercise adherence [16].

Self-efficacy is an important part of promoting healthy behavior and a significant determinant influencing the initiation and maintenance of positive health behaviors [17, 18]. The concept of self-efficacy was first proposed in 1977 [19]. According to self-efficacy theory, if individuals believe that they can achieve results, then they become more active and in control of their lives. A high sense of individual self-efficacy can lead to positive health behaviors [19, 20]. A higher sense of self-efficacy indicates that individuals will exhibit good exercise adherence, while a lower sense of self-efficacy is regarded as an obstacle to ensuring effective rehabilitation [21].

A number of studies have shown that enhancing self-efficacy is helpful to patient self-management and promotes health-related behaviors and good outcomes. Related interventions have been successfully applied in people with coronary heart disease [22], diabetes [23], hypertension [24], stroke [25], arthritis [26], and joint replacement rehabilitation [27, 28]. Therefore, patients should believe in their ability to perform specific physical activities to improve their rehabiliation after THA. Previous investigations have shown moderate self-efficacy of Chinese patients after hip and knee arthroplasty [29, 30]. Nevertheless, studies in this field are scarce, the majority used small sample sizes, and self-efficacy has not been considered in rehabilitation program development, leading to substantial extension of rehabilitation times and unsatisfactory outcomes.

The theory of self-efficacy provides a good basis for nursing interventions for patients after THA. This study is inspired by the self-efficacy theory of Bandura, which proposes that enhancement of self-efficacy is beneficial for patient rehabilitation and for achieving optimal outcomes. To investigate this further, we developed a 6-month self-efficacy-enhancing intervention (SEEI), to support adherence to prescribed home-based exercise for patients following THA. The SEEI was developed in rigorous accordance with Bandura’s self-efficacy theory and is a complex intervention. That theory is here applied to rehabilitation after THA.

Our primary hypothesis is that adherence to the prescribed home exercise program in the 6 months after THA will be higher in the group receiving the SEEI compared with the group receiving usual care. Our second hypothesis is that the SEEI group will exhibit greater improvements in other outcomes (e.g., pain, function, health-related quality of life [QOL], anxiety and depression, and another measure of exercise adherence) than the control group.

## Methods

### Study design

**Fig. 1:**
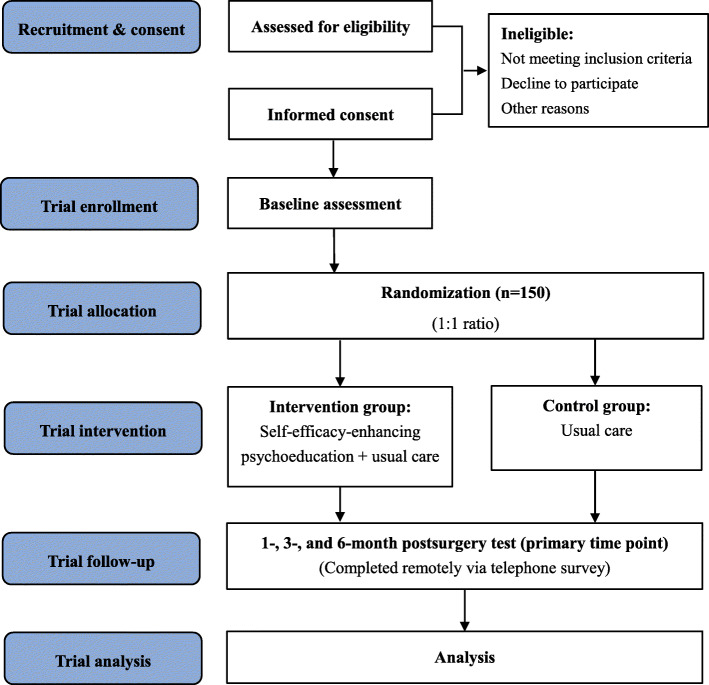
Flow diagram of the trial procedures

This prospective randomized controlled trial is to test the effect of a nurse-led SEEI, inspired by the theory of self-efficacy, and aimed to increase patient compliance following THA. All participants are randomly assigned to the SEEI group or usual care (control) group. This study will examine the effect of the intervention on patient exercise compliance after THA and evaluate the contribution of the intervention to the improvement of joint function. We hypothesize that participants in the experimental SEEI group will show significantly better exercise self-efficacy, a greater increase in exercise compliance, superior hip function, lower levels of anxiety and depression, and increased QOL. Data will be assessed at baseline and 1, 3, and 6 months after surgery. The study has been approved by the Ethics Committee of our institution and will be registered, constructed, and presented following the recommendations of the CONSORT statement [31] and SPIRIT guidelines [32] (see Fig. 1).

### Setting and population

The study setting is the Joint Surgery Unit of the Orthopedics Department at a hospital.

Patients on the surgical waiting list for THA will be invited to participate in the study after preoperative assessment. Men and women aged 18 years or older and scheduled for primary, single-side THA surgery at the Joint Surgery Unit are eligible for participation. Trial inclusion criteria are the following: participants must be able to read and understand Chinese, be willing to participate in a 6-month exercise intervention, and provide informed consent. Participants are ineligible if they meet one or more of the following exclusion criteria: (i) an unstable medical condition due to fracture, malignancy, infection, or THA failure; (ii) inability to provide informed consent due to mental disorder, dementia, or cognitive impairment that prevent completion of self-report surveys; (iii) co-existing conditions that would negate functional improvement with surgery and exercise (e.g., severe Parkinson disease, or hemiparesis); (iv) simultaneous bilateral THA; (v) surgery for neoplastic disease or imminently scheduled surgery and unavailable to complete the study procedures (e.g., bone tumor surgical operation); and (vi) planning another THA or total knee replacement within 6 months.

### Development of the SEEI program

#### Preparation phase

After reviewing international guidelines on the development of exercise interventions for adults undergoing hip surgery, the preparation of the SEEI program followed a three-stage process:

i) Setting up a nurse-led intervention team, mainly composed of orthopedic nursing clinical specialists, orthopedic surgeons, orthopedic physicians, senior orthopedic nurses, and research assistants, to control the overall research direction, formulate and evaluate intervention programs, and control the quality of research. A draft intervention was produced after a series of interviews with patients undergoing THA by experienced healthcare professionals; the specific content and protocol were subsequently validated by a panel of orthopedic experts.
ii) Formulating the “Handbook for Rehabilitation after discharge following THA” including two main aspects: post-discharge guidance, including general information about home reorganization, functional exercise, matters or follow-up needing attention, and daily life guidance during each rehabilitation period after THA; and a functional exercise diary in the rehabilitation manual that is convenient for patients to record their exercises and any problems. The functional exercise and daily life guidance sections contain illustrations and detailed written explanations. Contact information for the department, orthopedic specialist, and orthopedic nurse is at the back of the guide. The handbook will be available for patients in both groups.
iii) Registering a WeChat account for research and filming a functional exercise video. A WeChat account has been created specifically for the experimental group and is managed by a research assistant, as a convenient means to establish multiple contacts with patients and reduce follow-up loss. WeChat is used to promote exercise videos provided for patients and their families to learn about the various stages of joint replacement rehabilitation. WeChat will also be used to collect video data, including exercise and walking, during the rehabilitation process.

#### Intervention theoretical framework

The intervention is based on Bandura’s self-efficacy theory [33], combined with social cognitive theory (SCT) [34]. Social cognitive theory regards self-efficacy as an internal factor, influencing learner autonomy greatly by affecting learners’ goal setting, self-monitoring, self-evaluation, and strategy use [34]. Consistent with SCT, interventions target self-management knowledge, attitudes (self-efficacy), and behavioral abilities through a variety of printed materials and activities. The intervention program is also conducted according to Bandura’s self-efficacy theory [35], which includes four main aspects: individual past experience, witnessing the experiences of other individuals, verbal motivation from others, and physical and emotional support for patients, specific to the particular behavior [20]. The four strategies recommended by Bandura [36] have been incorporated into each component of the intervention. Individual past experience includes emphasizing the importance of functional exercise, helping participants to set achievable goals, encouraging them to observe and record their exercise behavior, and accumulating self-management experience according to their subjective physical experience. Vicarious experience includes sharing successful examples of patients who have experienced similar symptoms and recovered using self-management skills, to help participants build confidence, and encourage them to adhere to the functional exercise program. Verbal persuasion includes describing the benefits of exercise adherence to joint function, encouraging and acknowledging the participant’s ability to deal with symptoms, and helping those who encounter difficulties in finding specific causes and the following most suitable solutions. Physical and emotional support includes the explanation of possible symptoms during postoperative rehabilitation and the discussion of treatment strategies. Patients are supported in these four strategies during training sessions. Table 1 outlines examples of the four strategies [19].

**Table 1:** SEEI components, strategies, and techniques

Components	Strategies	Specific techniques
Individual past experience	• Setting achievable goals • Providing information on the benefits of functional exercise • Providing positive feedback	• Consulting with patients to develop functional exercise goals at different stages; making plans on when, where, and how to conduct regular physical activities • Providing information on the risk factors of a sedentary lifestyle and the advantages of functional exercise • Identifying postoperative rehabilitation challenges through discussion • Providing positive feedback on patient accomplishments
Vicarious experience	Sharing cases of successful rehabilitation	• Sharing previous success stories to build confidence • Introducing the successful experiences of others to encourage patients to complete physical activities in the following months
Verbal persuasion	Verbal encouragement, explanation, and persuasion	• Describing the benefits of physical activities • Asserting that patients have the ability to self-manage • Providing positive feedback on the patient’s efforts and giving verbal encouragement • Reinforcing the past and present successes or accomplishments of patients
Supporting patients physically and emotionally	• Developing strategies to cope with barriers • Help to seek social support	• Assessing patient expression of anxiety and depression • Identifying individual barriers to and resources for physical activity • Providing strategies for dealing with barriers and coping in the future (postsurgery; significance of social support)

#### Intervention structure and content

Challenges to successful post-THA functional achievement were identified, and strategies to address them were developed and integrated to design the intervention. Telephone interview was selected as the primary basis for the intervention because it is a low-cost, wide-coverage mechanism that can provide support intervention after THA for patients with limited mobility and transport difficulties [37]. Although this format has not been thoroughly investigated in patients with THA, it has been well-studied for patients with other conditions and used alone or in combination with face-to-face educational sessions and print materials [38, 39]. The initial intervention was further refined following a pilot pretest involving five patients.

The final intervention protocol includes a total of five sessions: 1 h of face-to-face education before discharge, and four additional sessions post-discharge between 1 and 6 months after surgery. Except for the hospital visit, all visits are telephone-based. The first call is approximately 40 min, with follow-up contacts ranging from 20 to 30 min. The intervention aims to enhance exercise adherence through the implementation of self-efficacy-enhancing strategies.

The initial face-to-face intervention is conducted in the hospital office before patients are discharged from the hospital after THA surgery. The focus of the initial session is to assess the functional exercise status and psychological condition of each individual, including the level of pain, the occurrence of complications, and knowledge of functional exercise. The nurse provides participants with the Rehabilitation Handbook after THA and instructs them to read it. The manual is used to supplement the face-to-face educational session. Participants are encouraged to refer to the manual at home for information. The nurses also provide video recordings of functional exercise after THA and teach patients the functional exercise movements and matters needing attention in daily life after surgery, as well as persuading and encouraging participants to set rehabilitation goals to be achieved 1 month post-THA, and mobilizing the encouragement and support of the participant’s family members.

During the 6-month follow-up period, four subsequent health-coaching sessions are conducted via telephone or the WeChat app. These sessions are designed to enhance participant compliance with functional exercises and are guided by a protocol. Participants in each follow-up are required to self-report the number of home exercises they have completed in the previous week, their level of pain, and their mental state. Nurses encourage and provide reinforcement of the participant’s efforts and success and empower them through support. Participants with a low adherence (<3 exercise sessions/week) are then asked to select a barrier from a scheduled list (forgot, too tired, injured, fear of movement, exercise inconvenience, lack of time, life stress) to explain why they were unable to complete the exercise as required. The nurse then provides a suggestion tailored to help overcome that barrier. According to the needs of each patient, the duration of each phone call ranges from 20 to 30 min.

Four senior orthopedic nurses with bachelor degrees or above and at least 10 years of orthopedic nursing experience have been selected in the target hospital. All nurses have orthopaedic specialist nurse certificates and are very familiar with the rehabilitation needs and physical and psychological problems of patients after THA. They are required to establish close relationships with the patients. Simultaneously, two research assistants are assigned to conduct the interventions with the patients, primarily through collection of questionnaire data, and assist with relevant intervention content. The two research assistants are very familiar with the care of patients undergoing joint replacement. Two days of training are provided for the nurses before intervention implementation, primarily including communication skills, consistency of the intervention program, strategies to encourage physiological and psychological changes, detailed descriptions of problems that occur in the rehabilitation process after THA, dos and don’ts for the telephone follow-up, and matters included in the intervention follow-up record form. All staff also receive training on the study protocol and the procedures for collecting informed consent and data assessment. The principal investigator will monitor the conduct of the intervention through observation sampling sessions.

### Assessment of outcomes

All assessment sessions (baseline [time 1], 1 month [time 2], 3 months [time 3], and 6 months [time 4] postsurgery) include measurement of the primary and secondary outcomes. A series of questionnaires related to health outcomes of the SEEI is implemented. During the baseline assessment, additional demographic information including health, medical history, and general health status is collected.

#### Primary outcomes

##### Self-efficacy for rehabilitation (SER) outcome scale

The 12-item SER was developed by Waldrop et al. [28], following Bandura’s guidelines [34] to assess participant beliefs about their ability to perform behaviors typical in physical rehabilitation for knee and hip surgery. The SER was developed in conjunction with rehabilitation psychologists and physical and occupational therapists. Items increase in difficulty (e.g., items assessing self-belief in ability to stretch a leg, to those assessing beliefs in one’s ability to walk). Items measure belief in ability to perform behaviors in varying therapy situations, including when experiencing pain and emotional distress. For each item, participants use an 11-point Likert scale, ranging from 0 (I cannot do) to 10 (certain I can do) to describe their confidence. The full score for the items is 120, and higher scores indicate more perceived self-efficacy for rehabilitation. According to Bandura [34], “efficacy scores are summed and then divided by the total number of items to indicate the strength of perceived self-efficacy for the activity domain.” Thus, mean self-efficacy scores were calculated. In this study, the reliability and validity of the Chinese version SER outcome scale will be assessed by six orthopedic nurses. The Cronbach’s alpha reliability coefficient value for the total scale was 0.942.

##### Functional exercise compliance scale for THA patients

The self-designed exercise compliance questionnaire was developed by Xinxin Li of Sun Yat-sen University [30]. It can be divided into three dimensions: physical exercise compliance, exercise monitoring compliance, and initiative-seeking advice compliance. Each item is rated on a 4-point scale (4 points = “always do,” 3 points = “almost always do,” 2 points = “occasionally do,” and 1 point = “never do”). The sum of item scores is the total score, with higher total scores indicating a greater level of compliance level with functional exercise. In this study, Cronbach’s alpha ranges from 0.915 to 0.947, with an intra-rater reliability value of 0.92.

#### Secondary outcome measures

##### Overall hip pain

Average overall hip pain in the past week is self-assessed using an 11-point numeric rating scale with score 0 representing “no pain” and 10 representing “extreme pain” (e.g., “pain as bad as you can imagine” or “worst pain imaginable”) [40–42]. Participants select the whole number (0–10) that best reflects the intensity of their pain.

##### Activity and participation (WHO-DAS II)

The World Health Organization Disability Assessment Schedule II (WHO-DAS II) was developed building on the theoretical models described and recommended by the 2001 International Classification of Functioning, Disability and Health, regarding the general evaluation and measurement of health conditions, disabilities, and psychometric variables [43]. The WHO-DAS II provides a standardized cross-cultural method for measuring the health and disability status of adults (aged over 18 years) over a 30-day period and contains 32 items covering six domains: cognition, mobility, self-care, getting along with people, activities of daily life, and social participation [44]. Scores are classified into five levels: no difficulty, mild difficulty, moderate difficulty, severe difficulty, and extreme difficulty [45]. The total score range is 0–100, with higher scores indicating higher limitations in daily life. In this study, the reliability of Cronbach’s alpha and intraclass correlation coefficient in the WHO-DAS II are 0.73–0.99 and 0.8–089, respectively, illustrating that the Chinese version is suitable to assess activity and participation [46].

##### Anxiety and depression

Anxiety and depression are measured using the Chinese version of the Hospital Anxiety and Depression Scale (C-HADS) [47]. The HADS is a self-report questionnaire designed to assess depression and anxiety and has been widely used among patient groups [48]. This instrument consists of 14 items (7 for anxiety [HAD-A] and 7 for depression [HAD-D]), which are used as two separate measures of psychological disturbance. Each subscale is scored from 0 to 21, with higher scores indicating greater distress. The psychometric properties of C-HADS have been confirmed, with satisfactory results in terms of reliability and validity [47]. In this study, the Cronbach’s alpha reliability coefficients for both the anxiety and depression subscales are both 0.85 [49].

##### Hip function

Hip function is monitored using the Harris Hip Score (HHS) for hip arthroplasty, a multidimensional disease-specific observational assessment of functioning, containing eight items representing pain, walking function, activities of daily living, and range of motion of the hip joint [50]. The HHS is widely used by physicians to assess physical function and pain relief in patients with hip disease [51]. The maximum score for the HHS is 100 points, with maximum possible scores for its components as follows: pain (44 points), function (47 points), range of motion (5 points), and deformity (4 points) [52]. A higher HHS score indicates better function.

##### QOL

Physical and mental health are assessed using the 12-item Short Form Health Survey (SF-12), a validated measure of QOL following joint replacement [53]. The SF-12 comprises 12 items for assessing physical and mental health and yields two summary scores: the physical and mental health composite scores [53, 54]. Scoring is norm-based, with a mean of 50 (SD = 10); lower SF-12 scores indicate poorer physical and/or mental health. The Chinese versions of SF-12 components are established as reliable and valid in older patients [55].

### Sample size, statistical analysis, and monitoring

#### Sample size calculation

The required sample size was determined as 64 patients in each group based on a previous study [56]. The study that our sample size calculations are based on experienced a low withdrawal rate (10%). We have made a conservative estimate of a 15% withdrawal rate; therefore, we intend to recruit 150 participants.

### Randomization and blinding

After completing all baseline data collection measures, participants are randomly divided into the intervention and control groups using computer-generated random numbers. Participants are assigned a series of continuous integers in the order of admission. To hide randomization, the independent researcher has prepared consecutively numbered, sealed, opaque envelopes, which are kept in a locked location. To avoid affecting exercise adherence behavior, participants are not informed of the two separate studies, re-randomization into this trial, or the exact details of the intervention. This process ensures adequate concealment, limiting the possibility of selection deviation. The statistician will be blinded to group allocation, which will be revealed upon completion of the statistical analyses.

### Data analysis

IBM SPSS 25.0 will be used to analyze the collected data, according to the intent-to-treat principle. An alpha level of 0.05 will be applied for all statistical tests, and a 95% confidence interval used for all estimated values. Missing data will be managed using the last-observation-carried-forward method; this imputation method replaces missing values in the post-baseline follow-up with the last observed value for each individual. Descriptive statistics and frequency distributions will be generated for patient demographic and disease-related characteristics. Independent *t* tests and *χ*^2^ analyses will be performed to evaluate whether there are significant differences in demographic or clinical characteristics and baseline measures between the control and intervention groups. A repeated measures multivariate analysis of variance will be used to test the effects of group assignments (intervention vs. control), time (pretest vs. posttests), and group by time interaction, for the four outcome measures. If the assumption of sphericity is not met, the degrees of freedom for the *F* ratio will be adjusted according to Greenhouse-Gessler epsilon. *P*-values < 0.05 will be considered statistically significant for all comparisons.

### Monitoring

The trial coordinator and lead investigators meet every 2 weeks to monitor adverse events and any issues relating to the trial and to review recruitment and trial progress.

## Discussion

This study protocol describes the theoretical basis and design of an ongoing randomized controlled trial that will test the effectiveness of a patient SEEI on THA functional outcomes. THA is a common and widely accepted treatment for advanced hip arthritis, and maximum improvement of joint function is a top priority. If effective, such interventions have great potential for dissemination. In addition to their potential to enhance patient rehabilitation self-efficacy, the lessons learned in this trial can provide guidance for tailored rehabilitation strategies after THA, including family activities and exercise programs.

### Limitations

The authors acknowledge the following limitations: this is a single-center trial with no cost-effectiveness analysis and limited patient and public involvement in the design of the intervention.

## Data Availability

Not applicable.

## Abbreviations

C-HADS: Chinese version of the Hospital Anxiety and Depression Scale
HHS: Harris Hip Score
QOL: Quality of life
SCT: Social cognitive theory
SEEI: Self-efficacy-enhancing intervention
SER: Self-efficacy for rehabilitation
THA: Total hip arthroplasty
WHO-DAS II: World Health Organization Disability Assessment Schedule II
